# Contribution of Genome-Wide Association Studies to Scientific Research: A Pragmatic Approach to Evaluate Their Impact

**DOI:** 10.1371/journal.pone.0071198

**Published:** 2013-08-14

**Authors:** Vito A. G. Ricigliano, Renato Umeton, Lorenzo Germinario, Eleonora Alma, Martina Briani, Noemi Di Segni, Dalma Montesanti, Giorgia Pierelli, Fabiana Cancrini, Cristiano Lomonaco, Francesca Grassi, Gabriella Palmieri, Marco Salvetti

**Affiliations:** 1 Centre for Experimental Neurological Therapies, (CENTERS) S. Andrea Hospital-site, Department of Neuroscience, Mental Health and Sensory Organs, NESMOS, “Sapienza”, University of Rome, Roma, Italy; 2 “Percorso di Eccellenza”, Faculty of Medicine and Psychology, “Sapienza”, University of Rome, Roma, Italy; 3 Department of Physiology and Pharmacology, “Sapienza”, University of Rome, Roma, Italy; 4 Department of Experimental Medicine, “Sapienza”, University of Rome, Roma, Italy; The Children’s Hospital of Philadelphia, United States of America

## Abstract

The factual value of genome-wide association studies (GWAS) for the understanding of multifactorial diseases is a matter of intense debate. Practical consequences for the development of more effective therapies do not seem to be around the corner. Here we propose a pragmatic and objective evaluation of how much new biology is arising from these studies, with particular attention to the information that can help prioritize therapeutic targets. We chose multiple sclerosis (MS) as a paradigm disease and assumed that, in pre-GWAS candidate-gene studies, the knowledge behind the choice of each gene reflected the understanding of the disease prior to the advent of GWAS. Importantly, this knowledge was based mainly on non-genetic, phenotypic grounds. We performed single-gene and pathway-oriented comparisons of old and new knowledge in MS by confronting an unbiased list of candidate genes in pre-GWAS association studies with those genes exceeding the genome-wide significance threshold in GWAS published from 2007 on. At the single gene level, the majority (94 out of 125) of GWAS-discovered variants had never been contemplated as plausible candidates in pre-GWAS association studies. The 31 genes that were present in both pre- and post-GWAS lists may be of particular interest in that they represent disease-associated variants whose pathogenetic relevance is supported at the phenotypic level (i.e. the phenotypic information that steered their selection as candidate genes in pre-GWAS association studies). As such they represent attractive therapeutic targets. Interestingly, our analysis shows that some of these variants are targets of pharmacologically active compounds, including drugs that are already registered for human use. Compared with the above single-gene analysis, at the pathway level GWAS results appear more coherent with previous knowledge, reinforcing some of the current views on MS pathogenesis and related therapeutic research. This study presents a pragmatic approach that helps interpret and exploit GWAS knowledge.

## Introduction

Genome-wide association screenings (GWAS) and, in a relatively near future, full-genome sequencing of large samples will substantially deepen our understanding of the etiology of multifactorial diseases, bringing new hope for the identification of definitive therapeutic targets. However, in spite of the spectacular technological progress that is making this happen, difficulties in the analysis and interpretation of the data are delaying the process [1]. Since the entity of this delay is unpredictable, it would be useful to look at the available data in a way that may help to set priorities in certain fields of clinical research.

An obvious strategy to assess the added value of the new knowledge that is being acquired is to confront it with the old one. Although successfully accomplished in other areas of bioinformatics [2], [3], this knowledge integration process has never been systematically and objectively attempted for GWAS data since the vast majority of genetic studies in the pre-GWAS era did not provide definitive evidence of associations, hence being non comparable. Nonetheless, being the bulk of the old studies based on a candidate-gene approach, irrespective of the reliability of their results the knowledge behind the choice of each gene is a faithful and thorough representation of pre-GWAS understanding of the disease.

We evaluated differences between pre- and post-GWAS knowledge in multiple sclerosis (MS). As first term of comparison, representing the pre-GWAS knowledge, we used an unbiased list of those candidate genes (included in GENOTATOR) [4] that had been considered appropriate choices for genetic studies based on pre-GWAS candidate-gene approach; as second term, we selected those genes exceeding the genome-wide significance threshold in GWAS published from 2007 on.

Based on the results of this analysis, performed in a single-gene and in a pathway-oriented approach, we evaluated the emergence of “black swans” from the GWAS data and the instances in which the old and the new knowledge reinforce each other. Importantly, such cases highlighted a potential coincidence between significant genetic variants and (endo)phenotypes of possible pathogenetic relevance, a particularly informative situation in that it tells us that the genetic association identified by GWAS may be coupled with pathogenetically relevant phenotypic variation. Being these variants attractive for pharmaceutical research, we also performed a survey of drugs that target the products of these genes including compounds that are already registered for human use and may be evaluated in proof-of concept clinical trials without further delay.

## Methods

To compare pre-GWAS knowledge with GWAS results we used two independent lists of genes. The first one, that we assume to be representative of pre-GWAS knowledge, contains all genes chosen as “candidate genes” for association studies in MS in the pre-GWAS era (all the studies included in GENOTATOR database and published up to august 2007). We obtained this list from the GENOTATOR meta-database [4] (http://GENOTATOR.hms.harvard.edu ). The second list is made of the genes that are reported as exceeding the threshold of genome-wide significance in the 15 GWAS published since 2007 on MS [5]–[19] (http://www.genome.gov/gwastudies/).

We compared the single gene composition of the two lists and then verified whether variations resulted in functional differences using Ingenuity Pathway Analysis (IPA). IPA settings included (1) strict experimentally-validated filter in the setting related to source data quality, (2) inclusion of information coming only from papers where tissues and cells belong to the following IPA categories: immune system, nervous system, and cell lines; (3) use only human-data and discard mouse and rat model data. Statistical significance was taken at p<0.05 (ie, -log(p)> = 1.3); B–H p-values denote p-values corrected for multiple testing using the Benjamini-Hochberg procedure (this technique relies on the fact that p-values are uniformly distributed under the null hypothesis) [20].

In IPA, the p-value associated with a function or a pathway in Global Functional Analysis (GFA) and Global Canonical Pathways (GCP) is a measure of the likelihood that the association between a set of focus genes in the experiment and a given process or pathway is due to random chance. The p-value is calculated using the right-tailed Fisher Exact Test. B–H correction method of accounting for multiple testing is used in this analysis, and enabled to control the error rate in our results and focused on the most significant biological functions associated with our genes of interest. A full mathematical and statistical explanation of the IPA procedure is available at http://www.ingenuity.com/wp-content/themes/ingenuitytheme/pdf/ipa/functions-pathways-pval-whitepaper.pdf.

Finally, we used the IPA software to find out all the molecules (pharmacologically active substances included) that directly or indirectly (connection mediated by a common interactor) interact with the products of the genes that compose our GENOTATOR and GWAS lists.

**Figure 1:**
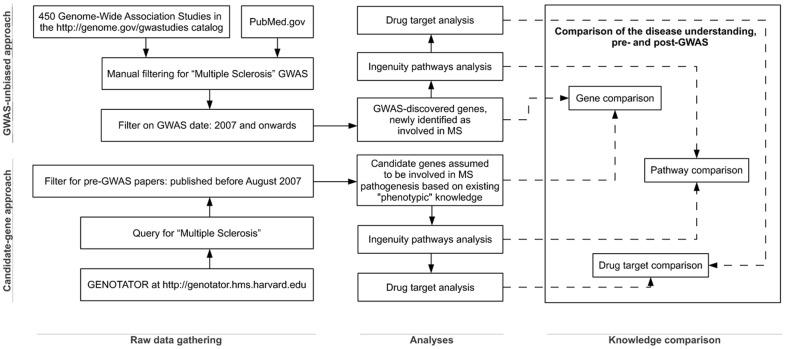
Study flow diagram. It summarizes of the methodology we designed and followed to compare the pre- and post-GWAS understanding of the disease by means of single gene analyses, pathway comparisons, and drug target evaluations.

The diagram in Figure 1 summarizes the methodology we designed and followed for our work of knowledge assessment and comparison.

## Results

Our analysis included 522 genes from GENOTATOR and 125 from GWAS, selected according to the parameters described in the Methods section (see also the diagram in Figure 1 for a snapshot of the study design). The GENOTATOR-derived panel can be taken as an unbiased representation of pre-GWAS, “phenotypic” knowledge (the conceptual background behind the choice of each “candidate” was mainly based on non-genetic information). The GWAS-derived panel reflects new information on the genetic variation that influences disease risk. The two panels were then confronted at the single-gene and at the pathway level.

**Figure 2:**
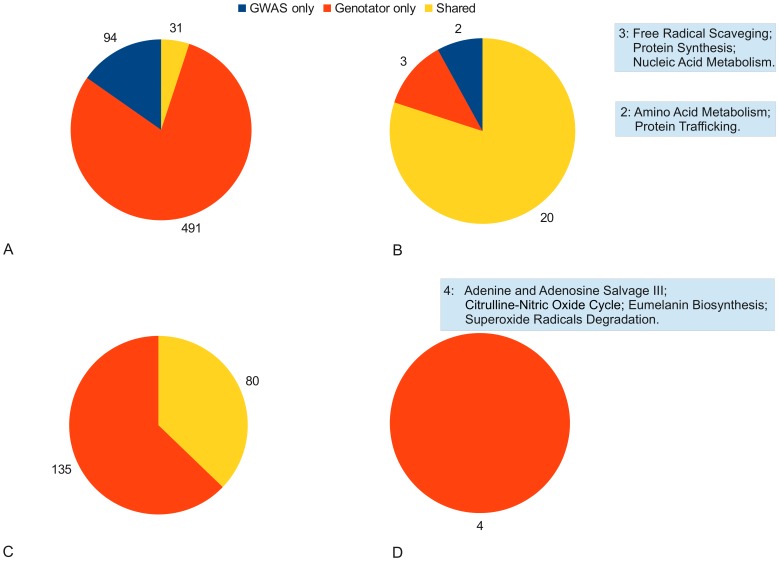
Comparison of GENOTATOR and GWAS gene lists. (**A**) results at the single-gene level; (**B**) results in terms of biological function derived from IPA analysis. Boxes describe specific biological functions; (**C**) signaling pathway comparison, resulting from IPA analysis; (**D**) comparison performed in terms of metabolic pathways, derived from IPA analysis. Box indicates “GENOTATOR-only” signaling pathways.

As shown in Fig. 2-A (and Table S1), at the single-gene level 31 genes upon the whole (647) could simultaneously be found in both GENOTATOR and GWAS lists, 491 were exclusive of the GENOTATOR list and 94 were exclusive of the GWAS list. This implies that 75.2% (94 out of 125) of the GWAS-discovered genes had never been considered as plausible candidates for single-gene association studies in MS. On the other hand the remaining 24.8% (31 out of 125) of the GWAS-identified genes confirm previous, phenotypic-derived knowledge.

Genes in the GENOTATOR and GWAS lists were then subjected to a pathway-oriented analysis in order to have a glance of the molecular and cellular functions associated to each test set. The Ingenuity analysis addressed the broader perspective of “biological function” first and then focused on “signaling pathways” and “metabolic pathways” (the only two categories contained in IPA canonical pathways) to obtain separate insight about specific cellular functions.

The “biological function” IPA showed a major overlap between the pre- (GENOTATOR data set) and post-GWAS knowledge (GWAS data set) (Fig. 2-B, Table S2 and Figure S1). In particular, GENOTATOR and GWAS data sets shared 20 out of 25 biological pathways. Of the 5 pathways that were exclusive of either data set, amino acid metabolism and protein trafficking emerged from GWAS data, whereas free radical scavenging, protein synthesis, nucleic acid metabolism emerged from GENOTATOR.

Comparison carried out at the signaling pathway level (Fig. 2-C, Table S3) showed a smaller overlap between the two data sets, as GENOTATOR and GWAS shared 80 pathways out of 215 (37.2%). Notably, in this case there was a considerable portion of pathways (135 upon the whole) emerging uniquely from GENOTATOR data.

The proportion of GENOTATOR pathways that were not confirmed in GWAS became preponderant in the “metabolic pathways” IPA, where no pathways were present in both GWAS and pre-GWAS lists of metabolic pathways (Fig. 2-D and Table S4).

**Figure 3:**
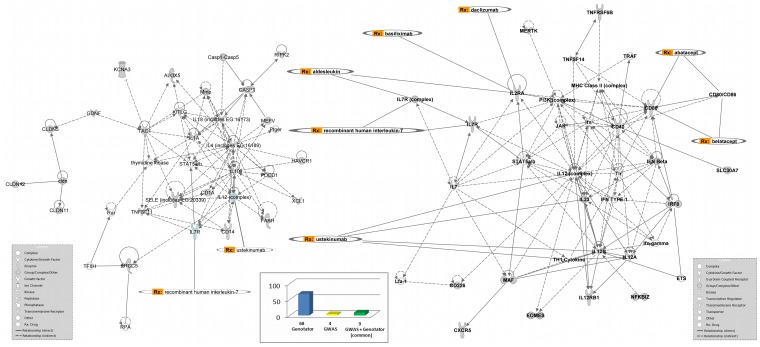
Results from the analysis of all the molecules directly or indirectly linked to GENOTATOR/GWAS lists of genes. Histogram chart (**center**) shows the absolute number of molecules contemporarily targeted by registered drugs or pharmacologically active compounds and also part of complex molecular networks involving GENOTATOR-only, GWAS-only, or common genes; (**left** and **right**): most significant molecular networks and related drugs.

To extract information that may steer the identification of “druggable” targets, we used the IPA software to find out all the molecules directly or indirectly interacting with the products of the genes in the GENOTATOR and GWAS lists. Among these, we focused our attention on those molecules (being either the original gene products or the associated proteins linked to them) that were targeted by registered drugs or by pharmacologically active (exogenous or endogenous) compounds and found that 9 (CD40, CD80, CD86, ESR1, HLA-DRB1, IL6, IL7R, IL12B, IL13) were genes present in both GWAS and GENOTATOR lists. Results of this analysis and the most significant networks, together with the related drugs, are described in Fig. 3 (and Table S5).

## Discussion

In principle, GWAS results are one of the best resources we can draw on for the development of new therapies in multifactorial diseases. Unfortunately their interpretation is neither simple nor granted [1]. Furthermore, the small effect size of the disease-associated variants discovered so far does not lend them to be considered as attractive therapeutic targets. However, the true pathogenetic role of these variants may erroneously appear limited, in the absence of comprehensive analyses of how this disease-relevant genetic variation correlates with functional/phenotypic knowledge. To provide conceptual support to the new information we confronted GWAS results with pre-GWAS, functional/phenotypic knowledge.

This comparison confirms, objectively, that GWAS are indeed broadening and refining our understanding of the genetic architecture of MS. The majority of the genes identified in GWAS are new with respect to those in the GENOTATOR list of pre-GWAS studies. Looking at the pathway-oriented analysis, in some instances (which were more frequent among “biological function”, less frequent among “signaling” and absent among “metabolic” pathways), the new knowledge strengthens hypotheses that had guided the selection of candidates for single-gene association analyses prior to the advent of GWAS; in others there are elements of novelty. Specifically, there are 2 biological pathways (amino acid metabolism and protein trafficking) that emerge only from GWAS data (according to IPA’s classification for bio- and canonical-pathways assessing the trajectory of a given knowledge dataset). Finally, the lack of overlap between GENOTATOR and GWAS knowledge at the “metabolic” IPA level may suggest a substantial denial of previous conjectures about the involvement of metabolic functions. Although this knowledge trajectory assessment contains, obviously, a publication bias (indeed, IPA’s knowledge repository is updated periodically with data coming from PubMed, KEGG, Gene Expression Omnibus, and all major scientific data repositories), our analysis can be repeated, for instance, every year, to update the trajectory where the GWAS research is overall headed.

The 31 genes that GWAS results have in common with pre-GWAS knowledge are of particular interest. In fact, in the pre-GWAS era, they had been selected based on non-genetic, phenotypic grounds. Therefore, functional information on the underlying biological processes is, to some extent, already available and, at least in some of these cases, they may represent *bona-fide* functional (endo)phenotypes [21], [22] whose pathogenetic relevance has been supported already. For these reasons genes such as CD40, CD5, CD80, CD86, CIITA, CXCR5, FCRL3, GALC, ICAM3, IL12A, IL12B, IL12RB1, IL6, IL7R, MAPK1, NFKB1, TNFRSF1A, may be considered foreground therapeutic targets (see Table S6 for functional information).

Among these, some are targeted by registered drugs and can therefore be placed even higher in an ideal ranking of interest. Nonetheless, pathogenetic relevance does not necessarily imply therapeutic efficacy. Additional parameters need to be taken into account in choosing the most appropriate therapeutic targets. In MS, the disappointing results of phase II clinical trials with Ustekinumab (CNTO 1275, Stelara®), a human monoclonal antibody targeting the interleukin (IL)-12/23 p40 subunit [23], may suggest that pleiotropic and redundant mediators of the immune response such as cytokines, while being pathogenetically relevant through processes that may last several years, are impractical targets for single therapies that ought to be effective in a relatively short time interval. Besides IL-12, and apart from CTLA4 (one published open-label phase 1 clinical trial of infusions of CTLA4Ig with positive immunologic effects [24] and one ongoing phase 2 study), there are no other completed or ongoing proof-of-concept trials on any of the 9 pathogenetically relevant molecules that may be targeted by registered drugs. The discussion of the issues that, if properly addressed, may help remove some roadblocks and facilitate repurposing trials goes beyond the scope of this study [25].

### Conclusions

Recent, citation metrics comparisons of pre-GWAS and GWAS publications have shown that GWAS are strong hypothesis generators [26]. Here, our comparison of pre-GWAS and GWAS results proposes a rational approach to the interpretation and exploitation of invaluable information such as that coming from GWAS, in MS and in other multifactorial diseases. It promises to become increasingly helpful as new genetic data and new data warehouses are available, particularly since it may contribute to prioritize the selection of therapeutic targets.

## Supporting Information

Figure S1 IPA line charts for each molecular and cellular function separately. X-axis indicates the group (GENOTATOR or GWAS), y-axis indicates the -log_10_(P value).
(TIFF)

Table S1 GENOTATOR-only and GWAS-only gene datasets.
(XLS)

Table S2 Biological-function comparative analysis for GENOTATOR and GWAS gene datasets.
(XLS)

Table S3 Signaling pathway comparative analysis for GENOTATOR and GWAS gene datasets.
(XLS)

Table S4 Metabolic pathway comparative analysis for GENOTATOR and GWAS gene datasets.
(XLS)

Table S5 Druggability extensive analysis for GENOTATOR-, GWAS-gene datasets and all the molecules that interact with the former two.
(XLS)

Table S6 Functional information on foreground therapeutic targets.
(DOCX)
