# Supplementary figures and images for: Contribution of Genome-Wide Association Studies to Scientific Research: A Pragmatic Approach to Evaluate Their Impact
Source: PLoS One. 2013 Aug 14;8(8):e71198. doi: 10.1371/journal.pone.0071198 (PMC3743868; doi:10.1371/journal.pone.0071198)

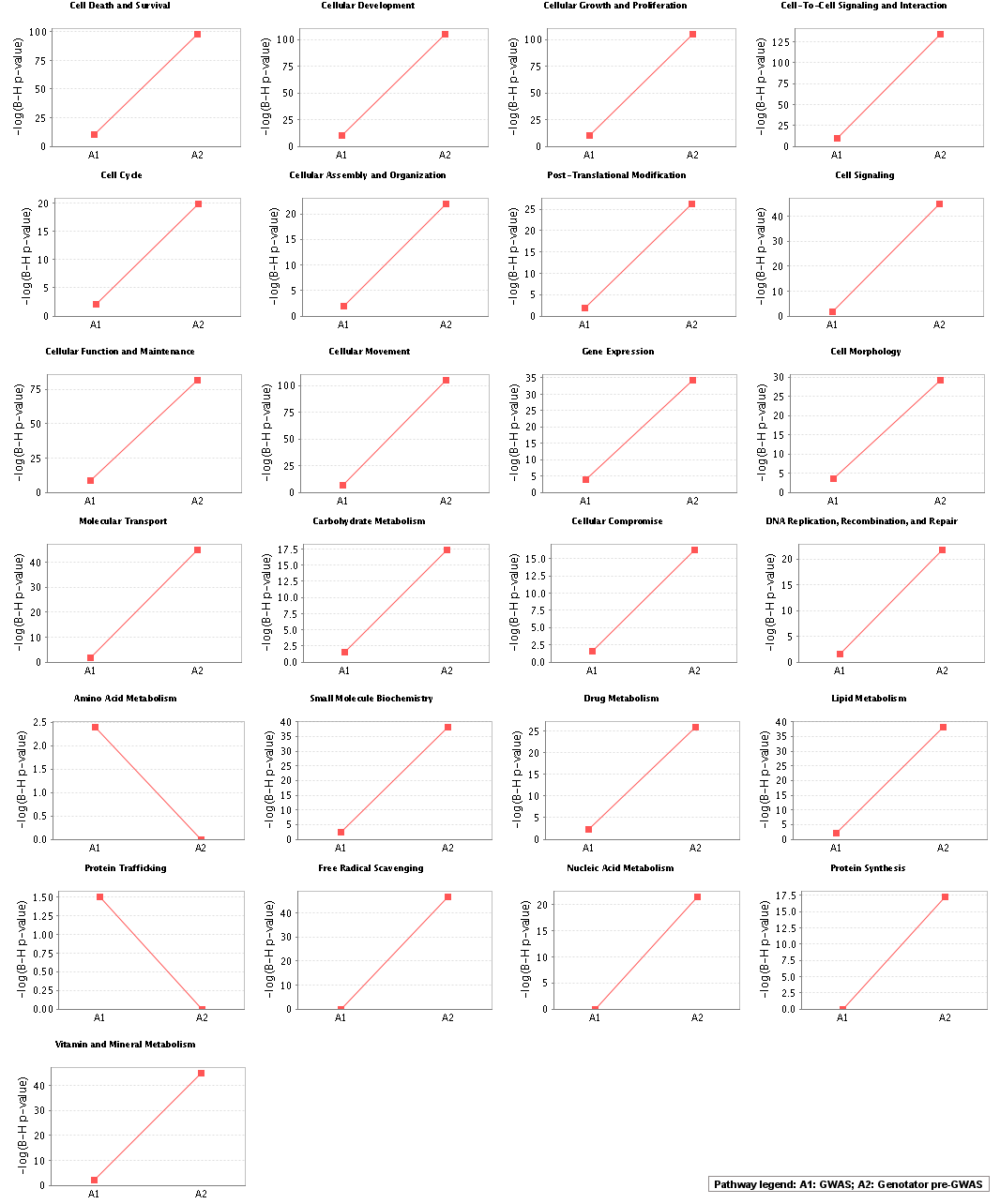

Supplement: Figure S1 — IPA line charts for each molecular and cellular function separately. X-axis indicates the group (GENOTATOR or GWAS), y-axis indicates the -log10(P value). (TIFF) [file pone.0071198.s001.tiff]
