# Supplementary material for: Contribution of Genome-Wide Association Studies to Scientific Research: A Pragmatic Approach to Evaluate Their Impact
Source: PLoS One. 2013 Aug 14;8(8):e71198. doi: 10.1371/journal.pone.0071198 (PMC3743868; doi:10.1371/journal.pone.0071198)
Supplement: Table S6 — Functional information on foreground therapeutic targets. (DOCX) [file pone.0071198.s007.docx]

| **Gene** | **Function^a^** | **Functional/Phenotypic variation in MS^b^** |
| --- | --- | --- |
| CD40 | CD40 interacts with the CD40 ligand, located primarily on T cells, playing a role in T cell-dependent immunoglobulin class switching, memory B-cell development, and other processes. | CD40-bearing cells (mostly macrophages or microglial cells) can be found in perivascular infiltrates of brain active lesions. An increased expression of CD40L^+^ cells has also been observed in the brains of patients [1, 2]. |
| CD5 | CD5 is a scavenger-like receptor expressed in association with the antigen-specific receptors on T and B-1a lymphocytes. | CD5 expression on B lymphocytes is significantly higher in patients with active MS when compared to patients with clinically stable disease.  High levels of CD5+ B-cells independently associate with increased risk of early conversion to MS in clinically isolated syndrome (CIS) patients [3, 4]. |
| CD80 | CD80 (or B7-1) provides co-stimulatory, regulatory signals for T lymphocytes as a consequence of binding to the CD28 and CTLA4 ligands on T cells. | The CD80/CD86-CD28/CD152 costimulatory interactions involved in the initiation, reactivation, and progression of the immunopathology in MS. Increased expression of B7-1 can be detected in perivenular inflammatory cuffs of acute MS plaques [5, 6]. |
| CD86 | Similarly to CD80, CD86 (or B7-2) delivers a second signal to T cells by interaction with CD28 or CTLA4. | CD86 expression is higher on monocytes derived from secondary progressive MS patients compared to those from relapsing-remitting MS or from healthy controls. Part of the therapeutic mechanism of IFNbeta may be to down-regulate CD86 expression [7]. |
| CIITA | CIITA is a positive regulator of class II major histocompatibility complex gene transcription. | CIITA variation increases MS risk, and this appears to depend on the presence of HLA-DRB1*1501 [8, 9]. |
| CXCR5 | CXCR5 is the receptor for the B-zone chemokine CXCL13 and it is involved in B-cell relocalization. | Increased CXCR5 levels can be found in primary progressive MS patients. Glatiramer acetate decreases the expression of this chemokine receptor [10, 11]. |
| FCRL3 | FCRL3 is a receptor for the Fc region (FcRs) of immunoglobulins. Such molecules modulate cellular and humoral immunity by linking their antibody ligands with effector cells of the immune system, and participate in autoimmune disorders. | Some variants of the Fc-receptor like-3 gene are involved in protection against MS, or in susceptibility to the disease [12, 13]. |
| GALC | GALC is a lysosomal enzyme involved in the catabolism of galactosylceramide, a major lipid in myelin, kidney and epithelial cells. | GALC substrates are major components of the myelin sheet [14]. |
| ICAM3 | ICAM 3 is an intercellular adhesion molecule and it is potentially the most important ligand for LFA1 in the initiation of the immune response. | ICAM3 expression is higher in cerebrospinal fluid and in brain lesions of MS patients [15, 16]. |
| IL12A  (p35) | Subunit in 2 distinct heterodimeric cytokines: interleukin-12 (IL12) and IL35. | Relapsing-remitting MS patients have increased expression of IL12A in whole blood [17]. |
| IL12B  (p40) | IL12B heterodimerizes with the IL12 p35 subunit (IL12A) to form IL12, and with the IL23 p19 subunit (IL23A) to form IL23. | IL12B polymorphisms are associated with MS. Serum levels are decreased during sustained treatment with IFN-beta1b [18, 19]. |
| IL12RB1 | IL12RB1 binds interleukin-12 with low affinity and is involved in IL12 transduction. When associated with IL12RB2 it forms a functional, high affinity receptor for IL12. It associates also with IL23R to form the interleukin-23 receptor. | The beta1 chain is part of the IL12 and IL23 receptor. IL23 may have a role in CNS inflammatory demyelination [20]. |
| IL6 | IL6 is a inducer of the acute phase response. It plays a role in the final differentiation of B-cells into Ig-secreting cells. Involved in lymphocyte and monocyte differentiation. | IL6 serum levels are higher in relapsing-remitting MS patients compared to controls. This molecule might reflect the global activity of the immune system in MS [21, 22]. |
| IL7R | IL7R is the receptor for interleukin-7, a glycoprotein involved in the regulation of lymphopoiesis. This ligand-receptor complex contributes to the normal development of T cells. | IL7R genotype influences susceptibility to MS. Increased soluble IL7Rα levels, as well as increased IL-7 levels, can be detected in patients with the predisposing IL7R variant [23, 24]. |
| MAPK1 | MAPK1/ERK2 and MAPK3/ERK1 are the 2 MAPKs which play an important role in the MAPK/ERK cascade. | The MAPK signaling cascade is upregulated in stress, immune response and autoimmune demyelinating disorders [25]. |
| NFKB1 | NF-kappa-B is a transcription factor present in almost all cell types; it is the endpoint of a series of signal transduction events related to immunity, inflammation, cell growth, differentiation, tumorigenesis. | NFKB pathway is upregulated in MS. Animal models demonstrate positive effects of attenuating central NFKB activation [26]. |
| TNFRSF1A | TNFRSF1A is a receptor for TNFSF2/TNFα. It contributes to the induction of cytocidal and non-cytocidal TNF effects. | Some polymorphisms in the gene (i.e. the amino acid substitution R92Q) influence MS risk and may increase proinflammatory signals [27, 28]. |

**a.** Function drawn from [http://omim.org](http://omim.org/) or [http://www.uniprot.org](http://www.uniprot.org/).

**b.** Arbitrary selection of studies and/or reviews that are representative of the prevailing view of the pathogenetic role in MS

**Supplementary Table 6 References**

1. Gerritse K, Laman JD, Noelle RJ, Aruffo A, Ledbetter JA, et al. (1996) CD40-CD40 ligand interactions in experimental allergic encephalomyelitis and multiple sclerosis. **Proc Nat Acad Sci USA** 93: 2499–2504.
2. Sokolova EA, Malkova NA, Korobko DS, Rozhdestvenskii AS, et al. (2013) Association of SNPs of CD40 Gene with Multiple Sclerosis in Russians. PLoS One 8: e61032.
3. [Seidi OA](http://www.ncbi.nlm.nih.gov/pubmed?term=Seidi%20OA%5BAuthor%5D&cauthor=true&cauthor_uid=12446024), [Semra YK](http://www.ncbi.nlm.nih.gov/pubmed?term=Semra%20YK%5BAuthor%5D&cauthor=true&cauthor_uid=12446024), [Sharief MK](http://www.ncbi.nlm.nih.gov/pubmed?term=Sharief%20MK%5BAuthor%5D&cauthor=true&cauthor_uid=12446024). (2002) Expression of CD5 on B lymphocytes correlates with disease activity in patients with multiple sclerosis. J Neuroimmunol 133: 205-10.
4. [Villar LM](http://www.ncbi.nlm.nih.gov/pubmed?term=Villar%20LM%5BAuthor%5D&cauthor=true&cauthor_uid=21436320), [Espiño M](http://www.ncbi.nlm.nih.gov/pubmed?term=Espi%C3%B1o%20M%5BAuthor%5D&cauthor=true&cauthor_uid=21436320), [Roldán E](http://www.ncbi.nlm.nih.gov/pubmed?term=Rold%C3%A1n%20E%5BAuthor%5D&cauthor=true&cauthor_uid=21436320), [Marín N](http://www.ncbi.nlm.nih.gov/pubmed?term=Mar%C3%ADn%20N%5BAuthor%5D&cauthor=true&cauthor_uid=21436320), et al. (2011) Increased peripheral blood CD5+ B cells predict earlier conversion to MS in high-risk clinically isolated syndromes. [Mult Scler.](http://www.ncbi.nlm.nih.gov/pubmed/21436320)  17: 690-4.
5. [Dudhgaonkar SP](http://www.ncbi.nlm.nih.gov/pubmed?term=Dudhgaonkar%20SP%5BAuthor%5D&cauthor=true&cauthor_uid=19917693), [Janardhanam SB](http://www.ncbi.nlm.nih.gov/pubmed?term=Janardhanam%20SB%5BAuthor%5D&cauthor=true&cauthor_uid=19917693), [Kodumudi KN](http://www.ncbi.nlm.nih.gov/pubmed?term=Kodumudi%20KN%5BAuthor%5D&cauthor=true&cauthor_uid=19917693), [Srinivasan M](http://www.ncbi.nlm.nih.gov/pubmed?term=Srinivasan%20M%5BAuthor%5D&cauthor=true&cauthor_uid=19917693). (2009) CD80 blockade enhance glucocorticoid-induced leucine zipper expression and suppress experimental autoimmune encephalomyelitis. J Immunol 183: 7505-13.
6. [Windhagen A](http://www.ncbi.nlm.nih.gov/pubmed?term=Windhagen%20A%5BAuthor%5D&cauthor=true&cauthor_uid=7500044), [Newcombe J](http://www.ncbi.nlm.nih.gov/pubmed?term=Newcombe%20J%5BAuthor%5D&cauthor=true&cauthor_uid=7500044), [Dangond F](http://www.ncbi.nlm.nih.gov/pubmed?term=Dangond%20F%5BAuthor%5D&cauthor=true&cauthor_uid=7500044), [Strand C](http://www.ncbi.nlm.nih.gov/pubmed?term=Strand%20C%5BAuthor%5D&cauthor=true&cauthor_uid=7500044). (1995) Expression of costimulatory molecules B7-1 (CD80), B7-2 (CD86), and interleukin 12 cytokine in multiple sclerosis lesions. J Exp Med 182: 1985-96.
7. [Filion LG](http://www.ncbi.nlm.nih.gov/pubmed?term=Filion%20LG%5BAuthor%5D&cauthor=true&cauthor_uid=12672403), [Matusevicius D](http://www.ncbi.nlm.nih.gov/pubmed?term=Matusevicius%20D%5BAuthor%5D&cauthor=true&cauthor_uid=12672403), [Graziani-Bowering GM](http://www.ncbi.nlm.nih.gov/pubmed?term=Graziani-Bowering%20GM%5BAuthor%5D&cauthor=true&cauthor_uid=12672403), [Kumar A](http://www.ncbi.nlm.nih.gov/pubmed?term=Kumar%20A%5BAuthor%5D&cauthor=true&cauthor_uid=12672403), [Freedman MS](http://www.ncbi.nlm.nih.gov/pubmed?term=Freedman%20MS%5BAuthor%5D&cauthor=true&cauthor_uid=12672403). (2003) Monocyte-derived IL12, CD86 (B7-2) and CD40L expression in relapsing and progressive multiple sclerosis. Clin Immunol 106: 127-38.
8. [Bronson PG](http://www.ncbi.nlm.nih.gov/pubmed?term=Bronson%20PG%5BAuthor%5D&cauthor=true&cauthor_uid=20211854), [Caillier S](http://www.ncbi.nlm.nih.gov/pubmed?term=Caillier%20S%5BAuthor%5D&cauthor=true&cauthor_uid=20211854), [Ramsay PP](http://www.ncbi.nlm.nih.gov/pubmed?term=Ramsay%20PP%5BAuthor%5D&cauthor=true&cauthor_uid=20211854), [McCauley JL](http://www.ncbi.nlm.nih.gov/pubmed?term=McCauley%20JL%5BAuthor%5D&cauthor=true&cauthor_uid=20211854). (2010) CIITA variation in the presence of HLA-DRB1*1501 increases risk for multiple sclerosis. Hum Mol Genet 19: 2331-40.
9. [Zuvich RL](http://www.ncbi.nlm.nih.gov/pubmed?term=Zuvich%20RL%5BAuthor%5D&cauthor=true&cauthor_uid=21653641), [Bush WS](http://www.ncbi.nlm.nih.gov/pubmed?term=Bush%20WS%5BAuthor%5D&cauthor=true&cauthor_uid=21653641), [McCauley JL](http://www.ncbi.nlm.nih.gov/pubmed?term=McCauley%20JL%5BAuthor%5D&cauthor=true&cauthor_uid=21653641), [Beecham AH](http://www.ncbi.nlm.nih.gov/pubmed?term=Beecham%20AH%5BAuthor%5D&cauthor=true&cauthor_uid=21653641). (2011) Interrogating the complex role of chromosome 16p13.13 in multiple sclerosis susceptibility: independent genetic signals in the CIITA-CLEC16A-SOCS1 gene complex. Hum Mol Genet 20: 3517-24.
10. [Furlan R](http://www.ncbi.nlm.nih.gov/pubmed?term=Furlan%20R%5BAuthor%5D&cauthor=true&cauthor_uid=15949850), [Rovaris M](http://www.ncbi.nlm.nih.gov/pubmed?term=Rovaris%20M%5BAuthor%5D&cauthor=true&cauthor_uid=15949850), [Martinelli Boneschi F](http://www.ncbi.nlm.nih.gov/pubmed?term=Martinelli%20Boneschi%20F%5BAuthor%5D&cauthor=true&cauthor_uid=15949850), [Khademi M](http://www.ncbi.nlm.nih.gov/pubmed?term=Khademi%20M%5BAuthor%5D&cauthor=true&cauthor_uid=15949850), et al. (2005) Immunological patterns identifying disease course and evolution in multiple sclerosis patients. J Neuroimmunol 165: 192-200.
11. [Begum-Haque S](http://www.ncbi.nlm.nih.gov/pubmed?term=Begum-Haque%20S%5BAuthor%5D&cauthor=true&cauthor_uid=21111489), [Christy M](http://www.ncbi.nlm.nih.gov/pubmed?term=Christy%20M%5BAuthor%5D&cauthor=true&cauthor_uid=21111489), [Ochoa-Reparaz J](http://www.ncbi.nlm.nih.gov/pubmed?term=Ochoa-Reparaz%20J%5BAuthor%5D&cauthor=true&cauthor_uid=21111489), [Nowak EC](http://www.ncbi.nlm.nih.gov/pubmed?term=Nowak%20EC%5BAuthor%5D&cauthor=true&cauthor_uid=21111489), et al. (2011) Augmentation of regulatory B cell activity in experimental allergic encephalomyelitis by glatiramer acetate. J Neuroimmunol 232: 136-44.
12. [Matesanz F](http://www.ncbi.nlm.nih.gov/pubmed?term=Matesanz%20F%5BAuthor%5D&cauthor=true&cauthor_uid=18313765), [Fernández O](http://www.ncbi.nlm.nih.gov/pubmed?term=Fern%C3%A1ndez%20O%5BAuthor%5D&cauthor=true&cauthor_uid=18313765), [Milne RL](http://www.ncbi.nlm.nih.gov/pubmed?term=Milne%20RL%5BAuthor%5D&cauthor=true&cauthor_uid=18313765), [Fedetz M](http://www.ncbi.nlm.nih.gov/pubmed?term=Fedetz%20M%5BAuthor%5D&cauthor=true&cauthor_uid=18313765), et al. (2008) The high producer variant of the Fc-receptor like-3 (FCRL3) gene is involved in protection against multiple sclerosis. J Neuroimmunol 195: 146-50.
13. [Martínez A](http://www.ncbi.nlm.nih.gov/pubmed?term=Mart%C3%ADnez%20A%5BAuthor%5D&cauthor=true&cauthor_uid=17617473), [Mas A](http://www.ncbi.nlm.nih.gov/pubmed?term=Mas%20A%5BAuthor%5D&cauthor=true&cauthor_uid=17617473), [de Las Heras V](http://www.ncbi.nlm.nih.gov/pubmed?term=de%20Las%20Heras%20V%5BAuthor%5D&cauthor=true&cauthor_uid=17617473), [Bartolomé M](http://www.ncbi.nlm.nih.gov/pubmed?term=Bartolom%C3%A9%20M%5BAuthor%5D&cauthor=true&cauthor_uid=17617473), et al. (2007) FcRL3 and multiple sclerosis pathogenesis: role in autoimmunity? [J Neuroimmunol](http://www.ncbi.nlm.nih.gov/pubmed/17617473) 189: 132-6.
14. [Blewett MM](http://www.ncbi.nlm.nih.gov/pubmed?term=Blewett%20MM%5BAuthor%5D&cauthor=true&cauthor_uid=17889444). (2008) Hypothesized role of galactocerebroside and NKT cells in the etiology of multiple sclerosis. Med Hypotheses 70: 826-30.
15. [Bö L](http://www.ncbi.nlm.nih.gov/pubmed?term=B%C3%B6%20L%5BAuthor%5D&cauthor=true&cauthor_uid=8858003), [Peterson JW](http://www.ncbi.nlm.nih.gov/pubmed?term=Peterson%20JW%5BAuthor%5D&cauthor=true&cauthor_uid=8858003), [Mørk S](http://www.ncbi.nlm.nih.gov/pubmed?term=M%C3%B8rk%20S%5BAuthor%5D&cauthor=true&cauthor_uid=8858003), [Hoffman PA](http://www.ncbi.nlm.nih.gov/pubmed?term=Hoffman%20PA%5BAuthor%5D&cauthor=true&cauthor_uid=8858003), et al. (1996) Distribution of immunoglobulin superfamily members ICAM-1, -2, -3, and the beta 2 integrin LFA-1 in multiple sclerosis lesions. J Neuropathol Exp Neurol 55: 1060-72.
16. [Kraus J](http://www.ncbi.nlm.nih.gov/pubmed?term=Kraus%20J%5BAuthor%5D&cauthor=true&cauthor_uid=11083511), [Oschmann P](http://www.ncbi.nlm.nih.gov/pubmed?term=Oschmann%20P%5BAuthor%5D&cauthor=true&cauthor_uid=11083511), [Engelhardt B](http://www.ncbi.nlm.nih.gov/pubmed?term=Engelhardt%20B%5BAuthor%5D&cauthor=true&cauthor_uid=11083511), [Stolz E](http://www.ncbi.nlm.nih.gov/pubmed?term=Stolz%20E%5BAuthor%5D&cauthor=true&cauthor_uid=11083511), et al. (2000) CD45RA+ ICAM-3+ lymphocytes in cerebrospinal fluid and blood as markers of disease activity in patients withmultiple sclerosis. Acta Neurol Scand 102: 326-32.
17. [Romme Christensen J](http://www.ncbi.nlm.nih.gov/pubmed?term=Romme%20Christensen%20J%5BAuthor%5D&cauthor=true&cauthor_uid=22978757), [Börnsen L](http://www.ncbi.nlm.nih.gov/pubmed?term=B%C3%B6rnsen%20L%5BAuthor%5D&cauthor=true&cauthor_uid=22978757), [Hesse D](http://www.ncbi.nlm.nih.gov/pubmed?term=Hesse%20D%5BAuthor%5D&cauthor=true&cauthor_uid=22978757), et al. (2012) Cellular sources of dysregulated cytokines in relapsing-remitting multiple sclerosis. J Neuroinflammation 9: 215.
18. [Alexander JS](http://www.ncbi.nlm.nih.gov/pubmed?term=Alexander%20JS%5BAuthor%5D&cauthor=true&cauthor_uid=20621951), [Harris MK](http://www.ncbi.nlm.nih.gov/pubmed?term=Harris%20MK%5BAuthor%5D&cauthor=true&cauthor_uid=20621951), [Wells SR](http://www.ncbi.nlm.nih.gov/pubmed?term=Wells%20SR%5BAuthor%5D&cauthor=true&cauthor_uid=20621951), [Mills G](http://www.ncbi.nlm.nih.gov/pubmed?term=Mills%20G%5BAuthor%5D&cauthor=true&cauthor_uid=20621951), et al. (2010) Alterations in serum MMP-8, MMP-9, IL-12p40 and IL-23 in multiple sclerosis patients treated with interferon-beta1b. Mult Scler 16: 801-9.
19. [Varadé J](http://www.ncbi.nlm.nih.gov/pubmed?term=Varad%C3%A9%20J%5BAuthor%5D&cauthor=true&cauthor_uid=22194214), [Comabella M](http://www.ncbi.nlm.nih.gov/pubmed?term=Comabella%20M%5BAuthor%5D&cauthor=true&cauthor_uid=22194214), [Ortiz MA](http://www.ncbi.nlm.nih.gov/pubmed?term=Ortiz%20MA%5BAuthor%5D&cauthor=true&cauthor_uid=22194214), [Arroyo R](http://www.ncbi.nlm.nih.gov/pubmed?term=Arroyo%20R%5BAuthor%5D&cauthor=true&cauthor_uid=22194214), et al. (2012) Replication study of 10 genes showing evidence for association with multiple sclerosis: validation of TMEM39A,IL12B and CBLB [correction of CLBL] genes. Mult Scler 18: 959-65.
20. [Gran B](http://www.ncbi.nlm.nih.gov/pubmed?term=Gran%20B%5BAuthor%5D&cauthor=true&cauthor_uid=15581393), [Zhang GX](http://www.ncbi.nlm.nih.gov/pubmed?term=Zhang%20GX%5BAuthor%5D&cauthor=true&cauthor_uid=15581393), [Rostami A](http://www.ncbi.nlm.nih.gov/pubmed?term=Rostami%20A%5BAuthor%5D&cauthor=true&cauthor_uid=15581393). (2004) Role of the IL-12/IL-23 system in the regulation of T-cell responses in central nervous system inflammatory demyelination. Crit Rev Immunol 24: 111-28.
21. [Hautecoeur P](http://www.ncbi.nlm.nih.gov/pubmed?term=Hautecoeur%20P%5BAuthor%5D&cauthor=true&cauthor_uid=9478261), [Forzy G](http://www.ncbi.nlm.nih.gov/pubmed?term=Forzy%20G%5BAuthor%5D&cauthor=true&cauthor_uid=9478261), [Gallois P](http://www.ncbi.nlm.nih.gov/pubmed?term=Gallois%20P%5BAuthor%5D&cauthor=true&cauthor_uid=9478261), [Demirbilek V](http://www.ncbi.nlm.nih.gov/pubmed?term=Demirbilek%20V%5BAuthor%5D&cauthor=true&cauthor_uid=9478261), [Feugas O](http://www.ncbi.nlm.nih.gov/pubmed?term=Feugas%20O%5BAuthor%5D&cauthor=true&cauthor_uid=9478261). (1997) Variations of IL2, IL6, TNF alpha plasmatic levels in relapsing remitting multiple sclerosis. Acta Neurol Belg 97: 240-3.
22. [Kallaur AP](http://www.ncbi.nlm.nih.gov/pubmed?term=Kallaur%20AP%5BAuthor%5D&cauthor=true&cauthor_uid=23292766), [Oliveira SR](http://www.ncbi.nlm.nih.gov/pubmed?term=Oliveira%20SR%5BAuthor%5D&cauthor=true&cauthor_uid=23292766), [Colado Simão AN](http://www.ncbi.nlm.nih.gov/pubmed?term=Colado%20Sim%C3%A3o%20AN%5BAuthor%5D&cauthor=true&cauthor_uid=23292766), [Delicato de Almeida ER](http://www.ncbi.nlm.nih.gov/pubmed?term=Delicato%20de%20Almeida%20ER%5BAuthor%5D&cauthor=true&cauthor_uid=23292766), et al. (2013) Cytokine profile in relapsing‑remitting multiple sclerosis patients and the association between progression and activity of the disease. Mol Med Rep 7: 1010-20.
23. [Gregory SG](http://www.ncbi.nlm.nih.gov/pubmed?term=Gregory%20SG%5BAuthor%5D&cauthor=true&cauthor_uid=17660817), [Schmidt S](http://www.ncbi.nlm.nih.gov/pubmed?term=Schmidt%20S%5BAuthor%5D&cauthor=true&cauthor_uid=17660817), [Seth P](http://www.ncbi.nlm.nih.gov/pubmed?term=Seth%20P%5BAuthor%5D&cauthor=true&cauthor_uid=17660817), [Oksenberg JR](http://www.ncbi.nlm.nih.gov/pubmed?term=Oksenberg%20JR%5BAuthor%5D&cauthor=true&cauthor_uid=17660817), et al. (2007)Interleukin 7 receptor alpha chain (IL7R) shows allelic and functional association with multiple sclerosis. Nat Genet 39: 1083-91.
24. [Lundström W](http://www.ncbi.nlm.nih.gov/pubmed?term=Lundstr%C3%B6m%20W%5BAuthor%5D&cauthor=true&cauthor_uid=23610432), [Highfill S](http://www.ncbi.nlm.nih.gov/pubmed?term=Highfill%20S%5BAuthor%5D&cauthor=true&cauthor_uid=23610432), [Walsh ST](http://www.ncbi.nlm.nih.gov/pubmed?term=Walsh%20ST%5BAuthor%5D&cauthor=true&cauthor_uid=23610432), [Beq S](http://www.ncbi.nlm.nih.gov/pubmed?term=Beq%20S%5BAuthor%5D&cauthor=true&cauthor_uid=23610432), et al. (2013) Soluble IL7Rα potentiates IL-7 bioactivity and promotes autoimmunity. [Proc Natl Acad Sci U S A.](http://www.ncbi.nlm.nih.gov/pubmed/23610432)  Apr 22. [Epub ahead of print]
25. [Guo X](http://www.ncbi.nlm.nih.gov/pubmed?term=Guo%20X%5BAuthor%5D&cauthor=true&cauthor_uid=21064192), [Harada C](http://www.ncbi.nlm.nih.gov/pubmed?term=Harada%20C%5BAuthor%5D&cauthor=true&cauthor_uid=21064192), [Namekata K](http://www.ncbi.nlm.nih.gov/pubmed?term=Namekata%20K%5BAuthor%5D&cauthor=true&cauthor_uid=21064192), [Matsuzawa A](http://www.ncbi.nlm.nih.gov/pubmed?term=Matsuzawa%20A%5BAuthor%5D&cauthor=true&cauthor_uid=21064192), et al. (2010) Regulation of the severity of neuroinflammation and demyelination by TLR-ASK1-p38 pathway. EMBO Mol Med 2: 504-15.
26. [Brück W](http://www.ncbi.nlm.nih.gov/pubmed?term=Br%C3%BCck%20W%5BAuthor%5D&cauthor=true&cauthor_uid=22766690), [Pförtner R](http://www.ncbi.nlm.nih.gov/pubmed?term=Pf%C3%B6rtner%20R%5BAuthor%5D&cauthor=true&cauthor_uid=22766690), [Pham T](http://www.ncbi.nlm.nih.gov/pubmed?term=Pham%20T%5BAuthor%5D&cauthor=true&cauthor_uid=22766690), [Zhang J](http://www.ncbi.nlm.nih.gov/pubmed?term=Zhang%20J%5BAuthor%5D&cauthor=true&cauthor_uid=22766690), [Hayardeny L](http://www.ncbi.nlm.nih.gov/pubmed?term=Hayardeny%20L%5BAuthor%5D&cauthor=true&cauthor_uid=22766690), et al. (2012) Reduced astrocytic NF-κB activation by laquinimod protects from cuprizone-induced demyelination. Acta Neuropathol 124: 411-24.
27. [Caminero A](http://www.ncbi.nlm.nih.gov/pubmed?term=Caminero%20A%5BAuthor%5D&cauthor=true&cauthor_uid=22059991), [Comabella M](http://www.ncbi.nlm.nih.gov/pubmed?term=Comabella%20M%5BAuthor%5D&cauthor=true&cauthor_uid=22059991), [Montalban X](http://www.ncbi.nlm.nih.gov/pubmed?term=Montalban%20X%5BAuthor%5D&cauthor=true&cauthor_uid=22059991). (2011) Role of tumour necrosis factor (TNF)-α and TNFRSF1A R92Q mutation in the pathogenesis of TNF receptor-associated periodic syndrome and multiple sclerosis. [Clin Exp Immunol](http://www.ncbi.nlm.nih.gov/pubmed/22059991) 166: 338-45.
28. [Comabella M](http://www.ncbi.nlm.nih.gov/pubmed?term=Comabella%20M%5BAuthor%5D&cauthor=true&cauthor_uid=23624563), [Caminero AB](http://www.ncbi.nlm.nih.gov/pubmed?term=Caminero%20AB%5BAuthor%5D&cauthor=true&cauthor_uid=23624563), [Malhotra S](http://www.ncbi.nlm.nih.gov/pubmed?term=Malhotra%20S%5BAuthor%5D&cauthor=true&cauthor_uid=23624563), [Agulló L](http://www.ncbi.nlm.nih.gov/pubmed?term=Agull%C3%B3%20L%5BAuthor%5D&cauthor=true&cauthor_uid=23624563), et al. (2013) TNFRSF1A polymorphisms rs1800693 and rs4149584 in patients with multiple sclerosis. [Neurology](http://www.ncbi.nlm.nih.gov/pubmed/23624563) Apr 26. [Epub ahead of print]
